# The Concurrent and Longitudinal Contributions of Linguistic and Cognitive Skills to L2 Writing Quality

**DOI:** 10.3390/jintelligence14010011

**Published:** 2026-01-06

**Authors:** Aiping Zhao, Fangzhu Chen, Xiang Li

**Affiliations:** 1 School of Foreign Languages and Literature, Shandong University, Jinan 250100, China; aipingzhao@sdu.edu.cn (A.Z.); fangzhuchen-bella@outlook.com (F.C.); 2 Interdisciplinary Center, Shandong University, Jinan 250100, China

**Keywords:** second language writing quality, linguistic skills, cognitive skills, inference making, longitudinal study

## Abstract

Research on second language (L2) writing has primarily focused on linguistic skills, with limited attention to higher-order cognitive skills such as inference making. This study expands prior research by examining both concurrent and longitudinal effects of linguistic skills (vocabulary, grammatical knowledge, and morphological awareness) and inference making on L2 English writing quality among 135 Chinese high school English learners. Students’ linguistic skills, inference making, and writing were assessed in Grade 10 and Grade 11. Regression analyses showed that, in Grade 10, vocabulary, grammatical knowledge, and inference making significantly predicted writing quality, whereas in Grade 11, morphological awareness, grammatical knowledge, and inference making were significant predictors. Longitudinally, Grade 10 morphological awareness uniquely contributed to L2 writing quality in Grade 11 after controlling for the autoregressive effect of L2 writing quality in Grade 10. These findings highlight the key role of inference making in writing development and reveal that linguistic skills contribute to writing differently across grades. Pedagogically, the results underscore the importance of targeting grade-specific skills to support higher-quality English writing.

## 1. Introduction

As a central means of academic success, professional communication, and cross-cultural participation, effective second language (L2) writing is widely recognized as a crucial yet difficult skill to develop (Evans et al., 2015; M. Kim et al., 2021). L2 writing is a highly demanding task that draws on multiple linguistic and cognitive skills. Linguistic skills such as vocabulary, grammatical knowledge, and morphological awareness have received the most attention (e.g., McCutchen et al., 2022; Miralpeix & Muñoz, 2018; Trapman et al., 2018). These skills provide writers with the linguistic resources necessary to express ideas accurately and coherently, and they have therefore been treated as central to writing development (Asaad & Shabdin, 2021; Schoonen et al., 2011). By contrast, cognitive skills, especially higher-order cognitive skills such as inference making, have received less attention in L2 writing research, despite their centrality to the complex processes of planning, organizing, and revising text (Y.-S. G. Kim & Schatschneider, 2017). Inference making is particularly important to L2 writing, as it enables writers to construct coherence and expand beyond the explicit content available (Hayes, 1996). Nevertheless, empirical research directly examining the relationship between inference making and L2 writing quality remains scarce.

To address this gap, the present study investigates the concurrent and longitudinal contributions of both linguistic skills (vocabulary, grammatical knowledge, and morphological awareness) and the cognitive skill of inference making to L2 English writing quality among Chinese high school students. By examining these factors within one model across time, this study seeks to provide a more comprehensive understanding of how linguistic and cognitive resources support L2 writing quality.

### 1.1. Linguistic and Cognitive Factors Impacting Writing Quality

The central process of writing is formulation, i.e., translating nonverbal information into verbal information, in which writers draw heavily on their linguistic resources (e.g., vocabulary, grammatical knowledge, morphological awareness; McCutchen et al., 2014, 2022; Schoonen et al., 2011). Vocabulary is essential for translating ideas into words and sentences (Babayiğit, 2014). Regardless of the specific linguistic demands of a writing task, a rich vocabulary facilitates the expression of thoughts with clarity and precision (Schoonen et al., 2011). The impact of vocabulary on L2 writing quality has been extensively examined in prior literature (e.g., Babayiğit, 2014; M. Kim et al., 2021; Miralpeix & Muñoz, 2018; Pan, 2023; Stæhr, 2008; Tong et al., 2023; Yang et al., 2019). For instance, M. Kim et al. (2021) found that L2 vocabulary explained a considerable amount of variance in L2 writing among university L2 English learners. Tong et al. (2023) investigated Chinese university L2 English learners and observed that vocabulary significantly predicted writing quality, with overall lexical knowledge accounting for 50% of the variance in L2 writing performance. Likewise, in a study among Chinese university students of English as a foreign language (EFL), Pan (2023) found that vocabulary positively influenced the quality of a reading-writing integrated task, where participants were required to summarize and comment on a given text. Nevertheless, research examining the longitudinal effect of vocabulary on L2 writing remains limited, although Schoonen et al. (2011) found that vocabulary at Grade 8 did not predict L2 English writing at Grade 9 among Dutch students.

Effective writing requires organizing words into coherent, well-structured sentences. Grammatical knowledge, i.e., learners’ understanding and application of rules governing word and phrase combination, is essential for constructing meaning in writing. Grammatical knowledge enables writers to construct well-formed sentences and refine their texts in accordance with their intended meanings (Jones et al., 2013; Marjokorpi, 2023). Like vocabulary, grammatical knowledge has a crucial impact on writing proficiency and its development, as it equips writers with a repertoire of linguistic forms from which to choose, enhancing both their ability to control language use and expressive flexibility (Marjokorpi, 2023; Trapman et al., 2018). Prior studies have revealed the significant relationship between grammatical knowledge and L2 writing quality (e.g., Jones et al., 2013; Schoonen et al., 2003, 2011). In a study among 281 eighth-grade EFL students, Schoonen et al. (2003) reported a substantial correlation of .84 between grammatical knowledge and writing scores. In a two-year longitudinal investigation involving 389 eighth-grade EFL students, Schoonen et al. (2011) observed that grammatical knowledge was a concurrent predictor of L2 writing at both Grade 8 and Grade 9. However, when the autoregressive effect of Grade 8 writing was controlled, Grade 8 grammatical knowledge did not longitudinally predict L2 writing in Grade 9.

Morphological awareness, a type of metalinguistic knowledge, refers to the ability to recognize, reflect on, and flexibly manipulate the internal structure of words while applying word formation rules (Carlisle, 1995; M. Li & Kirby, 2015; Varga et al., 2022). Rather than serving merely as a lexical resource, morphological awareness supports multiple dimensions of writing by enabling learners to efficiently retrieve and manipulate words. Derivational morphological awareness, in particular, allows writers to shift between grammatical categories, fostering lexical flexibility and syntactic complexity in text generation and revision (Asaad, 2024; McCutchen et al., 2014, 2022; McCutchen & Stull, 2015; Zhang, 2021). While substantial evidence from first language (L1) contexts links morphological awareness to writing quality (e.g., McCutchen et al., 2014, 2022; McCutchen & Stull, 2015), research in L2 contexts remains limited. Focusing on L2 postgraduates, Asaad and Shabdin (2021) reported a significant correlation between morphological awareness and academic writing quality. A subsequent intervention study by Asaad (2024) demonstrated that explicit morphological awareness instruction effectively enhanced students’ L2 academic writing. Zhang (2021), in a one-year longitudinal study with Chinese EFL university students, found that morphological awareness was positively and concurrently related to L2 argumentative writing quality. Zhang (2021) also revealed that explicit morphological knowledge (about morpheme-meaning and morpheme form) longitudinally predicted L2 writing.

Linguistic skills alone do not ensure effective writing. Writing is a complex activity that entails the simultaneous operation of multiple processes—such as retrieving lexical items, constructing sentences, and establishing local and global coherence (McCutchen, 1996). To coordinate these processes and construct a coherent text, writers also need to engage in higher-order cognitive skills, such as inference making (Cain et al., 2003; Y.-S. G. Kim & Schatschneider, 2017). Inference making is important to writing as it helps writers to build local and global coherence by integrating propositions within a text to ensure thematic continuity (Cain et al., 2003; Currie & Cain, 2015; Y.-S. G. Kim & Schatschneider, 2017). Although its role in reading comprehension is well established (Y.-S. G. Kim, 2017), research examining its impact on writing quality remains limited. Existing studies in L1 contexts nevertheless highlight its importance: Cragg and Nation (2006) demonstrated that poor comprehenders with inference difficulties produced texts comparable in length and syntactic complexity to their peers but with weaker overall structure. Extending this evidence, Y.-S. G. Kim and Schatschneider (2017) and Y.-S. G. Kim and Park (2019) both reported that inference making was independently related to L1 writing quality among English-speaking children, even after accounting for other cognitive skills. Similarly, Zhao & Guo (2025) revealed that inference making significantly contributed to L1 writing among Grade 3 Chinese children. These findings suggest that inference making supports the integration of ideas and construction of coherent discourse. However, while substantial evidence supports this relationship in L1 writing (Y.-S. G. Kim & Park, 2019; Y.-S. G. Kim & Schatschneider, 2017), its contribution to L2 writing remains unexplored.

### 1.2. Purpose of This Study

Despite the growing body of research on the component skills of L2 writing, two issues remain insufficiently addressed. First, the role of higher-order cognitive skills particularly inference making, in L2 writing, has been limited. Second, existing studies rarely examine the longitudinal contributions of multiple linguistic and cognitive factors to L2 writing quality. To address these gaps, the present study investigates both the concurrent and longitudinal contributions of several important linguistic skills (vocabulary, grammatical knowledge, morphological awareness) and inference making to L2 writing quality. The following two research questions guided this study.

1. To what extent do linguistic skills (i.e., vocabulary, grammatical knowledge, and morphological awareness) and higher-order cognitive skills (i.e., inference making) contribute to L2 English writing quality of Chinese high school students?
2. How do these factors longitudinally predict L2 English writing quality of Chinese high school students?

## 2. Materials and Methods

### 2.1. Participants

A total of 135 students (79 females, Mage = 15.86, SD = 0.60) were followed from Grade 10 to Grade 11 in this longitudinal study. The participants were from four intact classes in a public senior high school in southwest China based on administrative feasibility and teacher collaboration. The school shares key characteristics with typical Chinese public high schools in terms of curriculum structure, student demographics, and instructional practices. All participants were native Chinese speakers with no overseas experience. By the beginning of the study, they had received approximately 6.5 years of English classroom instruction. When we collected the first wave of data, participants were estimated by their teachers to have a vocabulary size of 2000–2500 words, and their English proficiency at Level 4 of China’s Standards of English Language Ability (CSE), which roughly corresponds to Level B1 in the Common European Framework of Reference (CEFR). That is, the participants had an intermediate level of English proficiency. English writing instruction at the school primarily focused on practical writings, such as letters and notices, as well as narrative and argumentative essays, with an emphasis on composition structure, choice of vocabulary, grammar accuracy, and coherence. According to teachers, students were generally capable of composing 120–150-word texts. Informed consent was obtained from all adult participants (aged 18 or above) and the parents of minor participants prior to their participation in the study.

### 2.2. Instruments

Vocabulary: Participants’ vocabulary was assessed in both Grade 10 and Grade 11 using the updated Vocabulary Levels Test (VLT; Webb et al., 2017). The updated VLT adopts a matching format that requires participants to match definitions with corresponding words. According to participants’ proficiency, the 2000-word and 3000-word levels were selected for Grade 10 and Grade 11, respectively. The different levels were selected to match the lexical development outlined in the English Curriculum Standards for General Senior High Schools (Ministry of Education of the People’s Republic of China, 2020) and to ensure measurement sensitivity by avoiding ceiling or floor effects. Each cluster included six words (three targets and three distractors) and three definitions. Participants matched words to definitions, receiving one point per correct response. Reliability (Cronbach’s α) for 2000-word and 3000-word levels vocabulary tests was .74 and .77, respectively.

Grammatical knowledge: Participants’ grammatical knowledge was measured in both Grade 10 and Grade 11 with an error-correction task adapted from a national examination repository in China. The task contained 20 sentences, each with one error. They assessed a comprehensive range of formal language knowledge, encompassing morphosyntactic structures (e.g., inflectional ending, possessive marker, subject-verb agreement, word order, tense) and lexical-grammatical aspects (e.g., use of prepositions, pronouns, articles). This integrated design aligned with the common practice of employing composite measures to capture broad grammatical knowledge in writing research (e.g., Marjokorpi, 2023; Schoonen et al., 2003, 2011). Participants identified and corrected the error in each sentence, receiving one point per correct correction (maximum = 20).

To examine whether these items formed separable underlying constructs, an exploratory factor analysis (EFA) using principal axis factoring with Varimax rotation was conducted in Grade 10. The Kaiser–Meyer–Olkin (KMO) value was .74, and Bartlett’s test of sphericity was significant, χ^2^(190) = 389.82 (*p* < .001), confirming that the data were suitable for factor analysis (Field, 2009). The rotated solution extracted four factors (eigenvalues > 1.00), but the factor structure was highly fragmented: the first factor explained only 8.14% of the variance and cross-loadings above .30 were observed for multiple items, indicating a lack of coherent subdimensions. A similar pattern was found in Grade 11, where the KMO was .64 and Bartlett’s test χ^2^(190) = 387.42 (*p* < .001), and the first factor accounted for only 8.06% of the variance. These results indicate that the task did not cluster into distinct subcomponents but instead reflected a diffuse profile of interrelated grammatical abilities, supporting the use of a total score as an aggregate index of comprehensive grammatical knowledge. Reliability (Cronbach’s α) for grammatical knowledge measures in Grade 10 and Grade 11 was .75 and .73, respectively.

Morphological awareness: Participants’ morphological awareness was assessed in both Grade 10 and Grade 11 with a part-of-speech conversion task adapted from Carlisle’s (2000) Test of Morphological Structure (TMS). Each task consisted of 20 items, equally divided between decomposition (transforming derived forms into base forms, e.g., from dangerous to danger) and derivation (producing derived forms from base words, e.g., from permit to permission). The task targeted derivational morphological awareness specifically, given its established role in fostering lexical and syntactic complexity in writing (McCutchen & Stull, 2015). Participants were required to provide the correct form to complete each sentence, receiving one point per correct answer (maximum = 20). Reliability (Cronbach’s α) for the morphological awareness measure was .78 and .83, respectively.

Inference making: Participants’ inference making was measured in both Grade 10 and Grade 11 with inference tasks from an American educational resource repository. To ensure that the task specifically tapped into inference making, all tasks were translated into Chinese and carefully selected to avoid culture-specific content. Both tasks in Grade 10 and Grade 11 included eight passages, each followed by two or three short-answer inference questions, totaling 19 questions. Responses were evaluated based on an answer key and checked for logical coherence, with one point awarded per correct response (maximum = 19). For instance, in one of the passages, students were asked the question “What is the thing that Mr. Thomas did again”? Students need to infer from the description “Mr. Thomas sitting at the front porch in bathrobe and bunny slippers”, “his neighbor lending his cellphone to Mr. Thomas so that he can call his wife”, “his neighbor offering him to keep a key over his house” that Mr. Thomas locked himself out again. Reliability (Cronbach’s α) for the inference making task was .79 and .69, respectively. The modest reliability at Grade 11 may have attenuated its observed associations with other variables (Schmidt & Hunter, 1999), which warranted caution in interpreting the findings.

L2 writing: Participants’ writing was tested in both Grade 10 and Grade 11. At each time point, students completed two writing tasks designed by assessment experts based on the Gaokao (China’s college entrance examination) English examination syllabus. The writing task is a continuation writing task, frequently used by Chinese students in both daily practice and formal examinations. In each task, participants were required to read a narrative passage with the ending omitted and continue the story by writing two paragraphs (Wang, 2021). This task format was selected because it constitutes a major Gaokao writing component (alongside a shorter, 80-word practical writing task; National Education Examinations Authority, 2025), providing a more extended sample for robust assessment. Moreover, it provides common contextual constraints for all participants through the source text, helping standardize the input and elicit language production that more directly reflects their writing proficiency (Wang & Wang, 2015). Tasks were selected to ensure comparability in genre, text length, topic familiarity, and prompt type. In Grade 10, the first task narrated a story about a conceited boy defeated by a hard-working boy in a running race (310 words), and the second was about a farmer wrongly accused of murder (290 words). In Grade 11, the first task described a woman’s first experience of running a half marathon (323 words), and the second was about a visit from an uncle unfamiliar to the children (320 words).

Compositions were evaluated using the 6 + 1 Trait Writing Model of Instruction and Assessment (Education Northwest, 2021). We selected this rubric instead of the Gaokao rubrics for writing because, while it closely aligns with the Gaokao framework, its more detailed classification of dimensions allows for a more precise and comprehensive assessment of learners’ writing ability. The Gaokao rubrics include three core dimensions: content, language, and structure but also point out that teachers should consider conventions such as spelling and punctuation. The 6 + 1 rubrics comprise five dimensions: ideas, word choice, sentence fluency, organization, and conventions. The ideas dimension corresponds to the “content” dimension in the Gaokao rubrics, evaluating the logic of the plot and details; the word choice and sentence fluency dimensions correspond to the “language” dimension, evaluating lexical appropriateness and diversity, and variety of sentence structures. The organization dimension corresponds to the “structure” dimension, assessing text structure development and coherence. The conventions dimension corresponds to spelling and punctuation. Each dimension was rated on a 0–6 scale, with a maximum score of 30 points per task. Scores from the two tasks were summed to form an overall writing quality score at each time point. The two tasks showed strong associations within each wave (Grade 10: *r* = .825, *p* < .001; Grade 11: *r* = .844, *p* < .001), indicating that they measured a common underlying writing ability and supporting the aggregation of task scores into a single index.

All writing samples were rated by two raters who had both applied the same scoring rubric in previous studies. To establish a shared understanding of the rating criteria, the raters first collaboratively scored 30 compositions in Grade 10 (15 from each task) and another 30 in Grade 11 (15 from each task). They then discussed any discrepancies in their ratings and reached a unanimous agreement on the sample scores. They later independently scored the remaining compositions. Inter-rater reliability was calculated from these independently scored data using the intraclass correlation coefficient (ICC). A two-way random-effects model with absolute agreement indicated excellent reliability: ICC = .989 (95% CI [.985, .993], *p* < .001) in Grade 10 and ICC = .994 (95% CI [.991, .996], *p* < .001) in Grade 11. Any discrepancies in the independent scoring were subsequently resolved through discussion, and the consensus scores were used in subsequent analyses.

### 2.3. Procedure

Data were collected during regular class hours on two separate days. At the beginning of the second semester of Grade 10, participants completed the vocabulary, grammatical knowledge, morphological awareness, inference making, and writing tasks under supervision. One year later, at the beginning of the second semester of Grade 11, the same cohort completed parallel versions of the vocabulary, grammar, morphology, and inference tasks and writing tasks under similar conditions. All tasks were completed individually without access to external aids.

### 2.4. Data Analysis Strategy

Descriptive statistics were used to summarize the characteristics of each variable. Bivariate correlations were calculated to examine associations among variables. Multiple linear regression analyses were performed to examine the concurrent effects, and hierarchical multiple linear regression analyses were used to examine the longitudinal effects of linguistic and cognitive skills on L2 writing quality.

## 3. Results

Table 1 reports the descriptive statistics and bivariate correlations among all measures across the two time points. All variables were approximately normally distributed (−1.95 ≤ skewness ≤ .55; −.48 ≤ kurtosis ≤ 5.42) according to the acceptable range of skewness and kurtosis (Skewness < ±2, Kurtosis < 7, West et al., 1995). All linguistic skills, including vocabulary, grammatical knowledge, and morphological awareness, were moderately to strongly related to L2 writing quality at both time points (.43 ≤ *r* ≤ .67) and across time (.50 ≤ *r* ≤ .57). Inference making was moderately related to writing quality in Grade 10 (*r* = .39) and weakly related to writing quality in Grade 11 (*r* = .19). Inference making in Grade 10 was weakly related to writing quality in Grade 11 (*r* = .20). L2 writing quality across the two time points was strongly correlated (*r* = .68).

Before running the regression analyses, regression assumptions were evaluated. Variance inflation factor (VIF) values across predictors ranged from 1.08 to 2.06, indicating no multicollinearity problems. For residual normality, the vast majority of standardized residuals across all three regression models fell within the acceptable absolute range of less than 2.58 (Field, 2009). Only one case at Time 1 (standardized residual = 3.75) and one case in the longitudinal model (standardized residual = 3.92) exceeded the ±3.29 cutoff (Field, 2009). To assess whether these cases exerted undue influence on the regression estimates, influential case diagnostics were examined. The maximum Cook’s distance values for the three models were .12, .15, and .26, respectively, all well below the threshold of 1, confirming that no cases exerted undue influence (Cook, 1977). In addition, scatterplots of standardized residuals against standardized predicted values (ZPRED on the X-axis and ZRESID on the Y-axis) showed randomly dispersed patterns with no systematic structure, indicating homoscedasticity (Field, 2009; Osborne & Waters, 2002). Given these satisfactory diagnostics, all data were retained for subsequent analyses.

To examine the concurrent effects of linguistic and cognitive skills on writing quality, multiple regression analyses were conducted for each time point separately. As shown in Table 2, in Grade 10, vocabulary (β = .16, *p* = .023), grammatical knowledge (β = .47, *p* < .001), and inference making (β = .32, *p* < .001) were significant predictors of L2 writing quality, while morphological awareness was not a significant predictor (β = .14, *p* = .09). The included variables explained 59% of the variance, representing a large overall effect (Cohen, 1988). By contrast, in Grade 11, grammatical knowledge (β = .33, *p* < .001), morphological awareness (β = .34, *p* < .001), and inference making (β = .14, *p* < .05) significantly predicted L2 writing quality, whereas vocabulary did not (β = .05, *p* = .55). Together, these variables accounted for 42% of the variance, indicating a large overall effect (Cohen, 1988).

To further explore longitudinal effects, a hierarchical multiple regression analysis was conducted with Grade 11 L2 writing quality as the dependent variable (see Table 3). To control for the autoregressive effect, Grade 10 L2 writing quality was entered into the equation as a predictor before other Grade 10 linguistic and cognitive skills. As shown in Table 3, Grade 10 L2 writing quality was a significant predictor (β = .68, *p* < .001) of Grade 11 L2 writing quality, accounting for 46% of the variance, which constitutes a large effect (Cohen, 1988). After the autoregressive effect was entered, only morphological awareness showed a unique longitudinal contribution (β = .27, *p* = .003) to Grade 11 L2 writing quality. The four Grade 10 linguistic and cognitive skills (vocabulary, grammatical knowledge, morphological awareness, and inference making) together explained an additional 8% of the variance, representing a small effect beyond Grade 10 L2 writing quality (Cohen, 1988).

## 4. Discussion

This study aimed to examine the concurrent and longitudinal contributions of linguistic and cognitive skills (i.e., vocabulary, grammatical knowledge, morphological awareness, and inference making) to L2 writing quality among Chinese high school students. The findings revealed grade-specific patterns for the four included skills to writing quality: in Grade 10, vocabulary, grammatical knowledge, and inference making significantly predicted concurrent L2 writing quality. In Grade 11, vocabulary was not a significant predictor, while morphological awareness joined grammatical knowledge and inference making to become key predictors of L2 writing quality. Longitudinally, after controlling for the autoregressive effect of L2 writing quality in Grade 10, only Grade 10 morphological awareness uniquely predicted L2 writing quality in Grade 11. Below, these findings are discussed in light of the extant literature, with a focus on aligning with prior evidence and explaining observed discrepancies.

### 4.1. Concurrent Predictors of L2 Writing Quality

Vocabulary was as a significant concurrent predictor of L2 writing quality in Grade 10, consistent with prior research highlighting the critical role of vocabulary in L2 writing (e.g., Babayiğit, 2014; M. Kim et al., 2021; Miralpeix & Muñoz, 2018; Pan, 2023; Stæhr, 2008; Tong et al., 2023; Yang et al., 2019). Lexical richness is a core metric for evaluating L2 writing quality, and a robust vocabulary was likely to confer an advantage to L2 learners in composing (Yang et al., 2019). A rich and varied vocabulary enables writers to choose the exact words that convey their intended meaning and tone, thereby enhancing clarity and accuracy in their writing. Surprisingly, vocabulary did not significantly predict L2 writing quality in Grade 11. The lack of association between vocabulary and L2 writing quality in Grade 11 may be explained by its moderate correlation with morphological awareness (*r* = .58). Given this moderate correlation, it is possible that a substantial portion of the variance in Grade 11 L2 writing quality attributed to vocabulary is shared with morphological awareness, thereby reducing vocabulary’s unique contribution to L2 writing quality.

Grammatical knowledge significantly predicted concurrent L2 writing quality in both Grade 10 and Grade 11, highlighting its fundamental role in L2 writing. This finding is consistent with findings from earlier studies that underscored its importance in L2 writing (e.g., Jones et al., 2013; Schoonen et al., 2003, 2011). It suggests that rich grammatical knowledge (e.g., a rich and flexible repertoire of sentence structure, correct use of word order and anaphora) allows students to not only construct correct sentences but also connect pieces of text accurately, contributing to the clarity, accuracy, and sophistication of written texts (Schoonen et al., 2011). In addition, rich grammatical knowledge enables writers to consciously attend to language use and more easily detect grammatical errors (e.g., errors in subject-verb agreement, conjunction use) that may obscure meaning (Marjokorpi, 2023). Therefore, grammatical knowledge facilitates not only the translating of ideas into linguistic expressions, but also the revising of his own writing, making it a necessary skill for producing clear and concise writing (Schoonen et al., 2003).

Morphological awareness did not significantly predict L2 writing quality in Grade 10, despite a significant bivariate correlation. There are two possible reasons for this absence of a relationship. First, as noted earlier, vocabulary and morphological awareness were moderately correlated in Grade 10 (*r* = .56), suggesting that a substantial portion of the variance in L2 writing quality explained by morphological awareness may have overlapped with that explained by vocabulary. Second, the lack of relationship might be related to the instruction of participants. We collected the first wave of data in the middle of Grade 10, with most of their English learning experience coming from junior high school (Grades 7–9) in China. Chinese junior high school English curricula prioritize direct vocabulary translation and grammar drills over morphological instruction (Dai et al., 2024). As a result, even though these Grade 10 learners possess a certain level of morphological awareness, they may struggle to apply it effectively in text production. This aligns with Zhang’s (2021) notion of a “delayed effect” of morphological awareness in L2 writing. That is, transfer from the receptive ability at the word level to the productive ability at the discourse level is likely to be indirect and prolonged. Thus, compared with the L1 learners in McCutchen and Stull’s (2015) study or the L2 university students in Asaad and Shabdin’s (2021) study, these Grade 10 EFL learners may not yet be able to leverage their morphological awareness to enhance writing quality.

The finding that morphological awareness predicts L2 writing quality in Grade 11 aligns with Asaad and Shabdin (2021). By Grade 11, learners have likely accumulated more exposure to morphological structures (e.g., affixes, word families) through high school instruction, enabling them to apply this knowledge to writing. Morphological awareness enables writers to select contextually appropriate word forms (e.g., describe, description, descriptive) that suit specific grammatical and rhetorical functions, thereby improving lexical diversity and precision (M. Li & Kirby, 2015; Varga et al., 2022). Morphological awareness also supports syntactic complexity (e.g., manipulating word forms to fit sentence structures), which contributes to L2 writing by improving the sophistication and preciseness of the written texts (Asaad, 2024; Asaad & Shabdin, 2021; Varga et al., 2022).

A unique and important finding of the present study is that inference making is a significant concurrent predictor of L2 writing quality in both Grade 10 and Grade 11. This finding extends L1 evidence (Zhao & Guo, 2025; Y.-S. G. Kim & Schatschneider, 2017; Y.-S. G. Kim & Park, 2019) to the L2 context and underscores that its impact is evident across both grades. It also adds to the literature that inference making is not only essential to reading comprehension but also to writing (Currie & Cain, 2015; Y.-S. G. Kim, 2017). Inference making may contribute to writing in at least the following two ways. First, it allows writers to integrate information across sentences and paragraphs, enabling them to maintain thematic continuity of the written text. Second, when writers make inferences, they draw on background knowledge and contextual cues to fill gaps and anticipate readers’ needs. This process supports the development of ideas that are conceptually rich and logically organized, leading to more coherent and reader-friendly writing. For L2 English learners, weak inference making may lead to off-topic, incoherent, or illogical writing. Consistent with this view, Cragg and Nation’s (2006) observed that poor comprehenders with weak inference making produced structurally flawed text. Despite a weak bivariate correlation with writing quality (*r* = .19), inference making yielded a significant contribution (β = .14, *p* < .05) when linguistic skills were accounted for. This finding suggests that when the shared linguistic variance is controlled, inference making accounts for additional, unique variance in writing quality, reflecting its important role in building coherence and integrating ideas across a text (Cain et al., 2003; Y.-S. G. Kim & Schatschneider, 2017).

### 4.2. Longitudinal Predictors of L2 Writing Quality

We found that, after controlling for the autoregressive effect of L2 writing quality in Grade 10, morphological awareness was the only Grade 10 variable that predicted L2 writing quality in Grade 11. The longitudinal predictive power of morphological awareness was an important finding. This finding aligns with Varga et al. (2022), which proposes that morphological awareness shows increasingly strong associations with language-related achievements over time. It indicates that morphological awareness is a fundamental metalinguistic skill that supports writing performance. Specifically, it enables learners to independently expand vocabulary—by identifying word stems and affixes to deduce the meaning of new words—and refine syntactic complexity over time (Asaad & Shabdin, 2021; McCutchen & Stull, 2015; Varga et al., 2022; Wolter & Green, 2013). These abilities are associated with writing quality. By contrast, there was no longitudinal associations between vocabulary, grammatical knowledge, and inference making in Grade 10 and L2 writing quality in Grade 11. This finding indicates that much of the variance explained by the linguistic and cognitive skills in L2 writing quality in Grade 11 may already have been captured by L2 writing in Grade 10.

The present finding of a longitudinal association between morphological awareness and L2 writing diverges from Zhang (2021), which reported no such association among L2 university students. The discrepancy may stem from two factors. First, this study focused on high school learners, who are in a critical period of morphological development, unlike university students in Zhang (2021), who may have already reached a plateau in morphological skill acquisition. This developmental stage difference makes high school learners more responsive to the long-term effect of morphological awareness on writing. Second, our study employed a different type of writing task from Zhang (2021). Specifically, we used a continuation writing task where there is a strong link between input (source text) and output (students’ written text). By contrast, Zhang (2021) adopted an independent writing task where there is only a task requirement (i.e., topic to write on). As noted by Zhang (2021), L2 learners often lack sufficient language input and output, limiting the benefits of emerging morphological awareness. The continuation writing task we adopted closely integrated language input and output, effectively compensating for L2 learners’ input-output deficits. This integration has likely activated morphological awareness more effectively: learners could apply morphological knowledge to recognize word structures in the input text and appropriately incorporate comparable structures in their own writing to fit their communicative needs, thereby enhancing both lexical selection and syntactic variety (Asaad & Shabdin, 2021; Carlisle, 1995; McCutchen & Stull, 2015). For Grade 11 learners, in particular, a year of targeted instruction and practice with continuation writing may have strengthened their ability to leverage morphological awareness, amplifying its longitudinal association with writing quality.

### 4.3. Limitations, Future Research, and Educational Implications

Several limitations of this study warrant acknowledgment. First, although the present study employed a longitudinal design, it included only two time points. As a result, any significant relationships observed should not be interpreted as causal in nature. Future longitudinal studies with three or more waves and experiment studies are needed to examine developmental trajectories and causal relationships. Second, the use of a convenient sample of intact classes from a single school limits the generalizability of the findings and leads to potential classroom or teacher effects. Given the small number of classes (four) and teachers, multilevel modeling was not feasible. Future research employing multilevel modeling with larger and more diverse samples is needed to confirm the present results. Third, the current study focused on narrative L2 writing tasks and used a composite score for overall writing quality instead of sub-scores for different dimensions. Future research should examine whether the observed patterns generalize to other writing types (e.g., argumentative, expository writing) and whether there are differential relations between the contributing factors and different dimensions of writing. Fourth, assessment of vocabulary and morphological awareness was limited to receptive vocabulary and derivational morphological awareness, leaving productive vocabulary and inflectional and compound morphological awareness unexamined (Tong et al., 2023; Varga et al., 2022; Yang et al., 2019). Consequently, the observed relationships of these skills with writing quality are specific to the measured dimensions, and their generalizability to the full constructs requires further investigation. Future studies are recommended to use more comprehensive measures that include different types of vocabulary and morphological awareness. Fifth, measurement choices might have imposed constraints. Although using different frequency bands to assess vocabulary at two time points was pedagogically justified, it limited cross-wave comparability and the interpretation of developmental change in the role of vocabulary over time. Similarly, the grammatical knowledge task was a composite measure encompassing morphosyntactic and lexical-grammatical aspects. Future studies would benefit from employing the same vocabulary measure across waves and using more focused assessments to better isolate the specific contribution of grammatical knowledge. Lastly, the current investigation did not account for other variables potentially pertinent to writing quality, such as basic reading skills, attention, working memory, and perspective-taking (Caemmerer et al., 2024; M. Kim et al., 2021; Y.-S. G. Kim & Schatschneider, 2017; S. Li, 2023). Future research incorporating these variables will be essential to extend the present findings.

Despite these limitations, the findings of this research offer targeted educational insights for L2 writing instruction in Chinese high schools. Given the grade-specific concurrent contributions and longitudinal role of the assessed linguistic and cognitive skills, tailoring instruction to strengthen these competencies is key to enhancing students’ L2 writing quality.

First, the significant concurrent impact of vocabulary on L2 writing quality in Grade 10 highlights the need for intentional, targeted vocabulary instruction. Teachers should strategically select appropriate words to teach rather than ask students to rote-memorize generic word lists (Tong et al., 2023). When teaching selected vocabulary, teachers should not only explicitly emphasize form-meaning links but also expand to related aspects such as collocations and synonyms. This deeper lexical knowledge enables students to use words more accurately and flexibly in their writing (Stæhr, 2008). Teachers are also suggested to incorporate varied activities, such as contextual word-guessing, word-pair association tasks, and pre-writing vocabulary activities that activate task-related vocabulary items and thus reduce cognitive load during the writing process (Webb, 2005; Yang et al., 2019). Furthermore, teachers can add timed vocabulary retrieval drills to strengthen learners’ flexible access and use of words during composition (Tong et al., 2023).

Second, the sustained concurrent contribution of grammatical knowledge across both grades calls for explicit and context-integrated teaching approaches, moving beyond mere rule transmission to foster students’ correct use of grammar. Specifically, teachers can design activities that guide students to analyze grammatical issues from not only grammatical rules taught in class but also real language use (Wijnands et al., 2021). Instead of isolated grammar drills, integrating grammar instruction into specific writing tasks can reinforce the application of grammatical knowledge in real composing scenarios (Jones et al., 2013). To further strengthen this contextual integration, teachers can incorporate tasks where students first analyze grammatical structures in authentic texts they read, and then apply this analytical awareness to refine the language in their own drafts (Marjokorpi, 2023).

Third, the notable longitudinal contribution of morphological awareness underscores the importance of embedding its development into the high school curriculum from the start, with consistent attention to its role in writing. Beginning in Grade 10, teachers can employ targeted instruction on word affixes and roots (e.g., breaking down complex words to analyze their morphemic components) to foster morphological awareness (Nation & Bauer, 2023; Varga et al., 2022). Additionally, morphological awareness instruction should be embedded within broader vocabulary, reading, and writing activities (e.g., analyzing morphemes in reading texts, applying word formation rules in writing drafts) to ensure it is not treated as an isolated skill (McCutchen et al., 2022).

Lastly, the concurrent contribution of inference making in both grades highlights the importance of designing pedagogical activities that promote inference making skills. Teachers can guide students to identify and use implicit and explicit contextual cues to build logical inferences (Cain & Oakhill, 1999). Importantly, teachers are encouraged to extend the instruction of inference-making skills beyond reading comprehension to writing. For example, after guiding students to infer implied meanings from a text, teachers might prompt them to consider how similar inferential processes can be applied when generating ideas, constructing arguments, or developing coherence in their own writing. In addition, leveraging textual structures like cause–effect and problem–solution to organize inference instruction can further help students deduce underlying themes or character motivations, which is critical for constructing coherent and logic writing (Rice et al., 2024).

Moreover, given that L2 writing quality in Grade 10 strongly predicted that of Grade 11, English teachers should prioritize and sustain writing instruction from the early stages of high school or earlier. This includes not only enhancing linguistic and cognitive skills but also fostering an educational environment that encourages writing development through scaffolded, progressively challenging tasks, in order to equip students with a robust foundation in L2 writing.

## 5. Conclusions

This study confirms the grade-specific and longitudinal roles of linguistic and cognitive skills in L2 writing quality among Chinese high school learners. Overall, the findings reveal a shift in the linguistic foundations of L2 writing: while vocabulary and grammatical knowledge underpin L2 writing quality in Grade 10, morphological awareness and grammatical knowledge are key predictors of L2 writing quality in Grade 11. Importantly, inference making remains as a significant contributor to L2 writing quality regardless of grades, highlighting its central role in L2 writing performance. The longitudinal effect of morphological awareness underscores its pivotal role in supporting L2 writing development. For teachers, these findings emphasize the need for nuanced, grade-specific instruction that targets both immediate (e.g., vocabulary, grammatical knowledge, inference making) and long-term (e.g., morphological awareness) predictors of L2 writing quality, thus ultimately supporting learners in achieving higher-quality L2 English writing.

## Figures and Tables

**Table 1:** Descriptive statistics and bivariate correlations among all measures across times.

Variable	1	2	3	4	5	6	7	8	9	10
1 Vocabulary (Grade 10)	-									
2 Grammatical knowledge (Grade 10)	.48 **	-								
3 Morphological awareness (Grade 10)	.56 **	.64 **	-							
4 Inference making (Grade 10)	.13	.12	−.07	-						
5 L2 writing quality (Grade 10)	.51 **	.67 **	.50 **	.39 **	-					
6 Vocabulary (Grade 11)	.42 **	.36 **	.34 **	.10	.33 **	-				
7 Grammatical knowledge (Grade 11)	.45 **	.58 **	.56 **	.16	.53 **	.52 **	-			
8 Morphological awareness (Grade 11)	.44 **	.49 **	.44 **	.05	.44 **	.58 **	.55 **	-		
9 Inference making (Grade 11)	.02	.04	.04	.28 **	.14	.08	.13	.02	-	
10 L2 writing quality (Grade 11)	.50 **	.55 **	.57 **	.20 *	.68 **	.43 **	.56 **	.55 **	.19 *	-
Range	4–18	2–20	1–19	2–19	13–57	3–18	1–18	0–20	6–19	12–60
M	12.96	13.29	13.39	12.65	36.48	12.52	10.16	7.79	16.73	34.71
SD	3.09	3.60	3.72	3.89	8.77	3.34	3.46	4.33	2.36	9.39
Skewness	−.63	−.91	−.65	−.42	−.46	−.71	−.19	.55	−1.95	.20
Kurtosis	.01	.70	−.04	−.48	.27	.23	−.27	−.19	5.42	−.29

Note: ** Correlation is significant at the .01 level (2-tailed). * Correlation is significant at the .05 level (2-tailed).

**Table 2:** Multiple regression analyses predicting L2 writing quality in Grade 10 and Grade 11.

	*β*	*t*	R^2^	F
Grade 10				
Vocabulary	.16 *	2.31	.59	46.22
Grammatical knowledge	.47 ***	6.18		
Morphological awareness	.14	1.69		
Inference making	.32 ***	5.50		
Grade 11				
Vocabulary	.05	.61	.42	23.15
Grammatical knowledge	.33 ***	3.89		
Morphological awareness	.34 ***	3.83		
Inference making	.14 *	2.04		

Note: * *p* < .05, *** *p* < .001.

**Table 3:** Hierarchical regression analysis predicting L2 writing quality in Grade 11.

Variable	*β*	*t*	R^2^	∆R^2^	∆F
Step 1			.46	.46	111.268 ***
L2 writing quality (Grade 10)	.68 ***	10.55			
Step 2			.54	.08	5.24 **
Vocabulary (Grade 10)	.11	1.41			
Grammatical knowledge (Grade 10)	.01	.06			
Morphological awareness (Grade 10)	.27 **	3.01			
Inference making (Grade 10)	.02	0.28			

Note: ** *p* < .01, *** *p* < .001.

## Data Availability

Data are available from the first author upon reasonable request.

## Abbreviations

The following abbreviations are used in this manuscript:

L2	Second language
EFL	English as a foreign language
L1	First language
CSE	China’s Standards of English Language Ability
CEFR	Common European Framework of Reference
VLT	Vocabulary Levels Test
TMS	Test of Morphological Structure
